# Sequence and analysis of a whole genome from Kuwaiti population subgroup of Persian ancestry

**DOI:** 10.1186/s12864-015-1233-x

**Published:** 2015-02-18

**Authors:** Gaurav Thareja, Sumi Elsa John, Prashantha Hebbar, Kazem Behbehani, Thangavel Alphonse Thanaraj, Osama Alsmadi

**Affiliations:** Dasman Diabetes Institute, P.O. Box 1180, Dasman, 15462 Kuwait

**Keywords:** Persian genome, Personal genome, Whole genome sequencing, Kuwaiti population, Arabian Peninsula

## Abstract

**Background:**

The 1000 Genome project paved the way for sequencing diverse human populations. New genome projects are being established to sequence underrepresented populations helping in understanding human genetic diversity. The Kuwait Genome Project an initiative to sequence individual genomes from the three subgroups of Kuwaiti population namely, Saudi Arabian tribe; “tent-dwelling” Bedouin; and Persian, attributing their ancestry to different regions in Arabian Peninsula and to modern-day Iran (West Asia). These subgroups were in line with settlement history and are confirmed by genetic studies. In this work, we report whole genome sequence of a Kuwaiti native from Persian subgroup at >37X coverage.

**Results:**

We document 3,573,824 SNPs, 404,090 insertions/deletions, and 11,138 structural variations. Out of the reported SNPs and indels, 85,939 are novel. We identify 295 ‘loss-of-function’ and 2,314 ’deleterious’ coding variants, some of which carry homozygous genotypes in the sequenced genome; the associated phenotypes include pharmacogenomic traits such as greater triglyceride lowering ability with fenofibrate treatment, and requirement of high warfarin dosage to elicit anticoagulation response. 6,328 non-coding SNPs associate with 811 phenotype traits: in congruence with medical history of the participant for Type 2 diabetes and β-Thalassemia, and of participant’s family for migraine, 72 (of 159 known) Type 2 diabetes, 3 (of 4) β-Thalassemia, and 76 (of 169) migraine variants are seen in the genome. Intergenome comparisons based on shared disease-causing variants, positions the sequenced genome between Asian and European genomes in congruence with geographical location of the region. On comparison, bead arrays perform better than sequencing platforms in correctly calling genotypes in low-coverage sequenced genome regions however in the event of novel SNP or indel near genotype calling position can lead to false calls using bead arrays.

**Conclusions:**

We report, for the first time, reference genome resource for the population of Persian ancestry. The resource provides a starting point for designing large-scale genetic studies in Peninsula including Kuwait, and Persian population. Such efforts on populations under-represented in global genome variation surveys help augment current knowledge on human genome diversity.

**Electronic supplementary material:**

The online version of this article (doi:10.1186/s12864-015-1233-x) contains supplementary material, which is available to authorized users.

## Background

Arabian Peninsula, situated at the nexus of Africa, Europe and Asia, has been implicated in early human migration route out of Africa and in early inter-continental trade routes [1-3]. The State of Kuwait is situated at northwestern tip of Persian Gulf and is bordered by Iraq and Saudi Arabia. Kuwaiti population is composed of early settlers from different regions in and around Arabian Peninsula namely Persia, Saudi Arabia and the deserts on the fringes of the Peninsula. Genetic features characterize Kuwaiti population into these three migratory subgroups [4-6].

The Persian subgroup is composed of people of West Asian (particularly Iranian) descent [7]. Iran, centrally located in the Asian continent, has served for centuries as gateway for movement of human population across diverse spheres of Asia and Europe. Iran is home to one of the major ancient civilizations and has rich cultural and social diversity [8]. Iranian population consists of diverse ethnic and linguistic groups namely Arabs, Armenians, Assyrians, Azeris, Baluchis, Gilaks, Mazandarani, Kurds, Lurs, Persian, Turkmen and Zoroastrians. Y-chromosome haplogroup studies also placed Iran at the nexus of tri-continental human migration and as a constant recipient of gene flow from the three continents [9,10]. The Persian subgroup of Kuwaiti population migrated into Kuwait from southwestern Iran and settled much before the establishment of modern-day Kuwait, the first group arriving around the second half of eighteenth century [7].

The 1000 Genomes Project [11] has illustrated that as high as 53% of rare variants (at minor allele frequency, MAF ≤ 0.5%) are observed only in individual populations, and that 17% of low-frequency variants (at MAF of 0.5% to 5%) are observed in single ancestry groups. Thus, it is essential to sequence diverse global populations, such as those that are underrepresented (e.g. Arabian Peninsula) in global genome-wide surveys, to enlarge our knowledge on human genome diversity.

Kuwait Genome Project (KGP) is an initiative to sequence individual genomes from three genetically distinct subgroups of Kuwaiti population at high coverage. In this paper, we report whole genome sequence of an individual from the Persian subgroup of Kuwaiti population (KWP1) at >37X coverage. We catalog a total of 3,573,824 single nucleotide polymorphisms (SNPs), 404,090 short insertions/deletions (indels) of length ≤ 50 bp, and 11,138 structural variations. The identified variants include deleterious and loss-of-function coding variants, as well as critical non-coding variants that are associated with phenotype traits. We illustrate how the medical history of the sequenced individual (Type 2 diabetes and β-Thalassemia) and of the family members (migraine) is reflected in the identified genome variants. The reported genome sequence, which can be used as ancestry specific genome for discovering additional variants [12], and genome variants add to the human variome diversity and may serve as terminus a quo for larger sequencing projects on this population group of Persian origin.

## Results

### Participant information and alignment statistics

A male participant aged 70 years, with self-reported history of Type 2 diabetes and β-Thalassemia (Minor) otherwise leading an active lifestyle was randomly selected from the Persian subgroup (Kuwait P as reported in our previous study [6]) of Kuwaiti population (Additional file 1: Figure S1). The participant’s surname lineage traces back its ancestry to a region in southern Iran and thus is in accordance with genetic clustering. The participant’s family is settled in Kuwait for at least 5 generations. Participant was further seen clustered with exomes of West Asian origin from the State of Qatar [13] (Additional file 2: Figure S2). The participant did not report any history of cardiovascular disorders or other major ailments.

A total of 1,111,992,216 (QC-passed) reads of 101 bps in length were generated covering human genome at 37.44X. Overall, 1,058,855,102 (95.22%) of the reads were mapped to reference human genome properly - *i.e.* the output of the Sequence Alignment/Map (SAM) tool for these mapped reads indicate that the reads aligned properly according to the aligner. Out of 1,058,855,102 mapped reads, 98.57% reads were mapped in proper pairs, 10,835,756 reads were singletons and 1,284,321 reads had their mates mapped to different chromosomes (mapQ > 5).

### Y-chromosome and mitochondrial haplogroups of the participant

The participant belongs to L1c [L-M357, L3] Y-chromosome haplogroup and HV14 [HV1a2] mitochondrial haplogroup. All three subgroups of L (L1a (M76), L1b (M317), and L1c (M357)) are present in Iran and Pakistan [14]. L1a is the most common subgroup found in India and L1b (M317) is rarest of the three in south Asia. L1c haplogroup is seen, though in low frequencies (2-4%), in the northern part of the Middle East (Iran, Turkey, Armenia, Kurdistan, Lebanon). It has been observed in 1.6% of the 938 male participants recruited to study Y-chromosome variation in modern Iran [10]. This haplogroup is also observed in Pashtun tribes from Afghanistan [15]. The HV mitochondrial haplogroup has a west Eurasian origin and is found throughout West Asia and Southeastern Europe, including Iran, Anatolia (present-day Turkey), the Caucasus Mountains of southern Russia and the Republic of Georgia [16]. Thus, the identified Y-chromosome and mitochondrial haplogroups are consistent with the Persian ancestry of the participant.

### Observed single nucleotide polymorphisms (SNPs) and indels

A total of 3,573,824 SNPs and 404,090 indels (of length ≤50 bp) were identified through comparing KWP1 genome with human reference genome (hg19) [17]. The number of reported SNPs is in concordance with mean of 3,596,151 SNPs as seen in sequencing 100 Malay Genomes using similar technology and at similar coverage [18].

**Figure 1:**
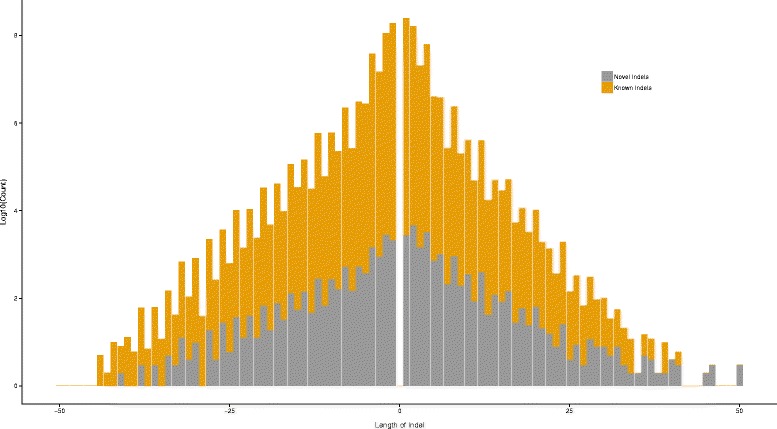
**Size distribution for indels in coding and non-coding regions.**

Of these variants, 58,186 (1.63%) SNPs and 27,753 (6.87%) indels are ‘novel’. This set of novel variants includes 1,547 SNPs and 4,577 indels having alleles not reported in dbSNP 138 [19]. The size distribution of the novel indels is similar to that of the known indels (Figure 1). The genome-wide Transition/Transversion ratio is 2.10 for known SNPs and 1.96 for novel SNPs (Additional file 3: Figure S3). The observed value in the case of novel SNPs at 1.96 is lower than the expected range of ~2.0-2.1; this can be due to one or a combination of the following reasons: possibility of residual false positives, possibility of a relative deficit in transitions due to sequencing context bias, or because of the apparently higher transition ratio often associated with low frequency variants [20].

### Annotation of SNPs and indels based on genomic locations

The identified SNPs and indels were classified into thirteen broad classes based on their genomic locations (Additional file 4: Table S1). In addition, the variants from coding regions were classified into thirteen classes based on their locations in exonic regions and their effects on protein sequences (Table 1). Most of the known and novel variants lie in intergenic regions.

**Table 1:** **Classification of the identified coding SNPs based on their location in exonic regions and their effects on protein sequences**

**Classification** ^**$**^	**Known SNPs**	**Novel SNPs**	**Known indels**	**Novel indels**
Init Codon	16	0	3	1
Non SNV	9197	193		
Splicing	68	2	44	
Stopgain	63	4	2	1
Stoploss	10	0		
Synonymous	10405	108		
Unknown	440	1	70	1
Del	-	-	84	3
FrameShift Del	-	-	42	8
FrameShift Ins	-	-	32	2
Ins	-	-	70	5

^$^Legends to the class types.

Splicing, Variant affects a nucleotide that is in a splicing region of a coding transcript.

Init Codon, Variant changes the start codon.

Frameshift Ins, An insertion that causes a shift in the codon reading frame.

Frameshift Del, A deletion that causes a shift in the codon reading frame.

Frameshift Sub, A substitution that causes a shift in the codon reading frame.

Stopgain, Variant causes a stop codon to be created at the variant site.

Stoploss, Variant changes a stop codon to something else.

Ins, An insertion that does not cause a frameshift.

Del, A deletion that does not cause a frameshift.

Sub, A substitution that does not cause a frameshift.

Nonsyn SNV, A single nucleotide variant that changes the amino acid produced by a codon.

Synonymous, A variant affecting 1 or more nucleotides that does not change the amino acid sequence.

Unknown, A problem was found with the protein coding sequence, See Invalid Transcripts.

SNP & Variation Suite v8.1 (SVS) [Bozeman, MT: Golden Helix, Inc] was used for classifications.

A set of 278 known exonic variants were annotated as LOF (Loss-of-Function) variants. This includes 147 SNPs and 131 indels which result in premature stops, splice-site disruptions and frame shifts. The number of identified LOF variants is consistent with previous reported average of 250 to 300 in 1000 Genomes Project [21]. Of these 278 exonic LOF variants, 130 variants (61 SNPs (Additional file 5: Table S2) and 69 indels (Additional file 6: Table S3)) are homozygous alternate leading to complete loss of function. Interestingly, none of the identified 17 novel exonic LOF variants (6 SNPs and 11 indels) are homozygous alternate. Previous studies have also shown at least 30 LOFs in homozygous state in a healthy individual [22]. A higher number of homozygous LOFs could be due to high inbreeding coefficient of 0.0196, as estimated in our previous study [6], and tradition of consanguineous marriages in the family of participant.

Allele frequency analysis, using data from 1000 Genomes Project, of 58 (out of the above-mentioned 61) homozygous exonic LOF SNPs showed that 13 of these SNPs are rare variants (with MAF ≤ 0.05) (Additional file 7: Figure S4); thus it can be safely inferred that these LOF mutations are genuine and are not sequencing artifacts. Furthermore, out of 61 LOF homozygous SNPs, 17 are annotated as expression quantitative trait loci (eQTLs) in various populations [23] (Additional file 8: Table S4). These 130 homozygous LOF variants lie in 125 genes. Clustering of these genes based on gene function using DAVID [24] did not result in any significant cluster classification.

We further report 2,123 known (Additional file 9: Table S5) and 91 novel SNPs (Additional file 10: Table S6) that are predicted to be “deleterious”; these deleterious SNPs are distributed across 1,173 genes. 6.6% of the novel deleterious SNPs and 28% of the known deleterious SNPs are homozygous, Of the known 2,123 deleterious SNPs, 9 are common with the LOF data set. There is no enrichment of disease classes, but analysis using DAVID tool showed an enrichment of olfactory receptor activity with Benjamin corrected p-value of 1.2E-19. A similar enrichment in olfactory receptor activity was observed in analysis of whole genome sequence of Indian individual [25].

### Associating identified variants with phenotype traits

A major challenge in delineating functional aspects from personal genomes lies in relating the variants with biological phenotypes such as pharmacogenomic traits, disease risks and other common phenotypic traits (e.g. hair and eye color). Of the identified homozygous LOF variants, rs5758511 [22:g.42336172G > A leading to stop codon gain] (*CENPM* gene [MIM: *610152] is seen associated with the biological phenotype of decrease in birth weight [26]. As birth weight is often associated with metabolic syndromes [27], it is important to look for these signals relatively early. Further, the *CENPM* gene has been shown to be associated with benzene haematotoxicity [28] and body weight [29].

We could further delineate genotype-disease associations in the case of 28 SNPs from the set of ‘known’ deleterious SNPs (Table 2). The participant is a carrier of homozygous genotypes at three of the following deleterious SNPs; rs2304256 [19:g.10475652C > A] [Val362Phe] (*TYK2* gene [MIM:*176941]), rs1799990 [20:g.4680251A > G] [Met129Val] (*PRNP* gene [MIM:*176640]), rs676210 [2:g.21231524G > A] [Pro2739Leu] (*APOB* gene [MIM:+107730] – these markers are associated with susceptibility to Type 1 diabetes [30], long-term memory [31] and triglycerides level [32-34], The observed homozygous genotype of (*AA*) at rs2304256 provides protection from Type 1 diabetes [30]. As regards to the homozygous genotype of *GG* seen at rs1799990 in the sequenced genome, it has been reported that twenty-four hours after a word list-learning task, carriers of either the (*AA*) or (*AG*) genotypes recalled 17% more information than (*GG*) genotype carriers, but short-term memory was unaffected [31]. rs676210 has been associated with triglyceride (TG) response to fenofibrate treatment for hypertriglyceridemia [35]; participants having the homozygous *AA* genotype (as is the case with the sequenced genome in this study) at rs676210 have greater triglyceride lowering ability than those with the *CC* genotype in response to fenofibrate treatment. Though risk alleles are seen at these variants, the manifestation is not seen in the participant. For example, participant medication history does not state use of fenofibrate and his triglyceride levels are reported to be normal at 0.82 mmol/L. In future, if these phenotypes manifests in the participant, these findings can aid physicians in treating the participant.

**Table 2:** **Genotype-phenotype associations in the case of 28 SNPs from the set of ‘known’ deleterious SNPs**

**SNP**	**Mapped gene [MIM]**	**Genotype**	**Phenotype**	**Pubmed ID**
rs1801133 [1:g.11856378G > A] [Ala263Val]	MTHFR [607093]	het	Homocysteine levels	23824729
rs676210 [2:g.21231524G > A] [Pro2739Leu]	APOB [107730]	hom	triglyceride (TG) response to fenofibrate treatment for hypertriglyceridemia; LDL (oxidized), Lipid metabolism phenotypes	23247145
rs6756629 [2:g.44065090G > A] [Arg50Cys]	ABCG5 [605459]	het	Cholesterol, total, LDL cholesterol	19060911
rs16891982 [2:g.21231524G > A] [Pro2739Leu]	SLC45A2 [606202]	het	Skin pigmentation, Hair color, Eye color	17999355
rs2043112 [5:g.38955796G > A] [Ser837Phe]	RICTOR [609022]	het	Obesity-related traits	23251661
rs30187 [5:g.96124330 T > C] [Lys528Arg]	ERAP1 [606832]	het	Ankylosing spondylitis	21743469
rs33980500 [6:g.111913262C > T] [Asp10Asn]	TRAF3IP2 [607043]	het	Psoriatic arthritis, Psoriasis	20953186
rs7076156 [10:g.64415184A > G] [Thr62Ala]	ZNF365 [607818]	het	Crohn’s disease	22412388
rs5006884 [11:g.5373251C > T] [Leu172Phe]	OR51B6 (Paralog of OR51E1 MIM:* [611267]	het	Fetal hemoglobin levels	20018918
rs11042023 [11:g.8662516 T > C] [His322Arg]	TRIM66 [612000]	het	Obesity	23563607
rs2306029 [11:g.46893108 T > C] [Ser1554Gly]	LRP4 [604270]	het	D-dimer levels	21502573
rs11230563 [11:g.60776209C > T] [Arg225Trp]	CD6 [186720]	het	Inflammatory bowel disease	23128233
rs6591182 [11:g.65349756 T > G] [Val538Gly]	EHBP1L1	het	Non-alcoholic fatty liver disease histology (lobular)	20708005
rs1042602 [11:g.88911696C > A] [Ser192Tyr]	TYR [606933]	het	Skin pigmentation, Freckles	17999355
rs1126809 [11:g.89017961G > A] [Arg402Gln]	TYR [606933]	het	Tanning,Sunburns	23548203
rs3213764 [12:g.14587301A > G] [Lys530Arg]	ATF7IP [613644]	het	Prostate-specific antigen levels	23359319
rs4149056 [12:g.21331549 T > C] [Val174Ala]	SLCO1B1 [604843]	het	Sex hormone-binding globulin levels, Bilirubin levels, Response to statin therapy	22829776
rs883079 [12:g.114793240C > T] [3' UTR variant]	TBX5 [601620]	het	Ventricular conduction	21076409
rs17730281 [15:g.53907948G > A] [Leu829Phe]	WDR72 [613214]	het	Renal function-related traits (BUN)	22797727
rs12968116 [18:g.55322502C > T] [Arg952Gln]	ATP8B1 [602397]	het	Liver enzyme levels (gamma-glutamyl transferase)	22001757
rs2304256 [19:g.10475652C > A] [Val362Phe]	TYK2 [176941]	hom	Type 1 diabetes, Type 1 diabetes autoantibodies	21829393
rs2108622 [19:g.15990431C > T] [Val433Met]	CYP4F2 [604426]	het	Acenocoumarol maintenance dosage, Vitamin E levels, Metabolite levels, Warfarin maintenance dose, Response to Vitamin E supplementation	19578179
rs8100241 [19:g.17392894G > A] [Ala20Thr]	ANKLE1	het	Breast cancer	22976474
rs2363956 [19:g.17394124 T > G] [Leu173Trp]	ANKLE1	het	Ovarian cancer	20852633
rs1434579 [19:g.44932972C > T] [Gly662Arg]	ZNF229 [Paralog of ZNF224 MIM:* [194555]	het	Tuberculosis	20694014
rs2303759 [19:g.49869051 T > G] [Met34Arg]	DKKL1 [605418]	het	Multiple sclerosis	21833088
rs1799990 [20:g.4680251A > G] [Met129Val]	PRNP [176640]	hom	Long-term memory	19081515
rs738409 [22:g.44324727C > G] [Ile148Met]	PNPLA3 [609567]	het	Liver enzyme levels (alanine transaminase),Nonalcoholic fatty liver disease	22001757

Only in the case of these 28 (of the identified 2123 ‘known’ deleterious SNPs), the genotype-phenotype associations are known in NHGRI GWAS Catalog.

The remaining deleterious SNPs that are heterozygous, include rs2108622 [19:g.15990431C > T] [Val433Met] (*CYP4F2* gene [MIM: *604426]) that is associated with Warfarin drug response and altered Vitamin K (VK1) metabolism. It has been shown that compared to individuals with the homozygous *CYP4F2* genotype (*CC*) at rs2108622, carriers of the heterozygous *CT* (and homozygous *TT*) genotypes require higher warfarin doses to elicit anticoagulation response [36-39]. An earlier study in Kuwait has also reported poor quality of anticoagulation with Warfarin [40]. Other phenotypes such as multiple sclerosis and obesity are also seen in this list of deleterious SNPs (Table 2).

From the remaining known non-coding SNPs, we annotated 6,328 SNPs corresponding to 811 traits (Additional file 11: Table S7). 2,457 (38.8%) of these 6,328 SNPs are homozygous. In congruence with the reported history of Type 2 diabetes and β-Thalassemia (Minor) by the participant, we find the presented genome to exhibit 72 out of 159 Type 2 diabetes and 3 out of 4 β-Thalassemia variants as annotated in NHGRI GWAS Catalog. We find that most of the observed 72 Type 2 diabetes SNPs have been discovered in populations of South and East Asian descent [41] (19 in South Asian, 7 in South & East Asian (Study sample contains participants from South and East Asian populations), and 18 in East Asian populations). As single locus has a very modest effect on Type 2 Diabetes susceptibility and all the reported loci seem to partly account for the heritability of Type 2 Diabetes, it is difficult to say whether one or few or all of these 72 SNPs influence the phenotype in the individual (Additional file 12: Table S8). The three β-Thalassemia variants observed in the sequenced genome are: rs766432 [2:g.60719970C > A] (seen as homozygous in the studied genome; intronic, *BCL11A* gene [MIM: *606557]) [42], rs9376092 [6:g.135427144C > A] (intergenic, *HBS1L* [MIM:*612450]-MYB [MIM: *189990] genes) [43], and rs2071348 [11:g.5264146 T > G] (intronic; *HBBP1* gene) [44,45]. It has been reported that these three loci are the best and common predictors of the disease severity in β-Thalassemia [43]. Further, it is noted that there is incidence of migraine in the family of the participant; in conformity with this, we find the presented genome to exhibit 76 out of 169 migraine variants annotated in GWAS Catalog.

### Comparing the sequenced genome with individual genomes from other continents

**Figure 2:**
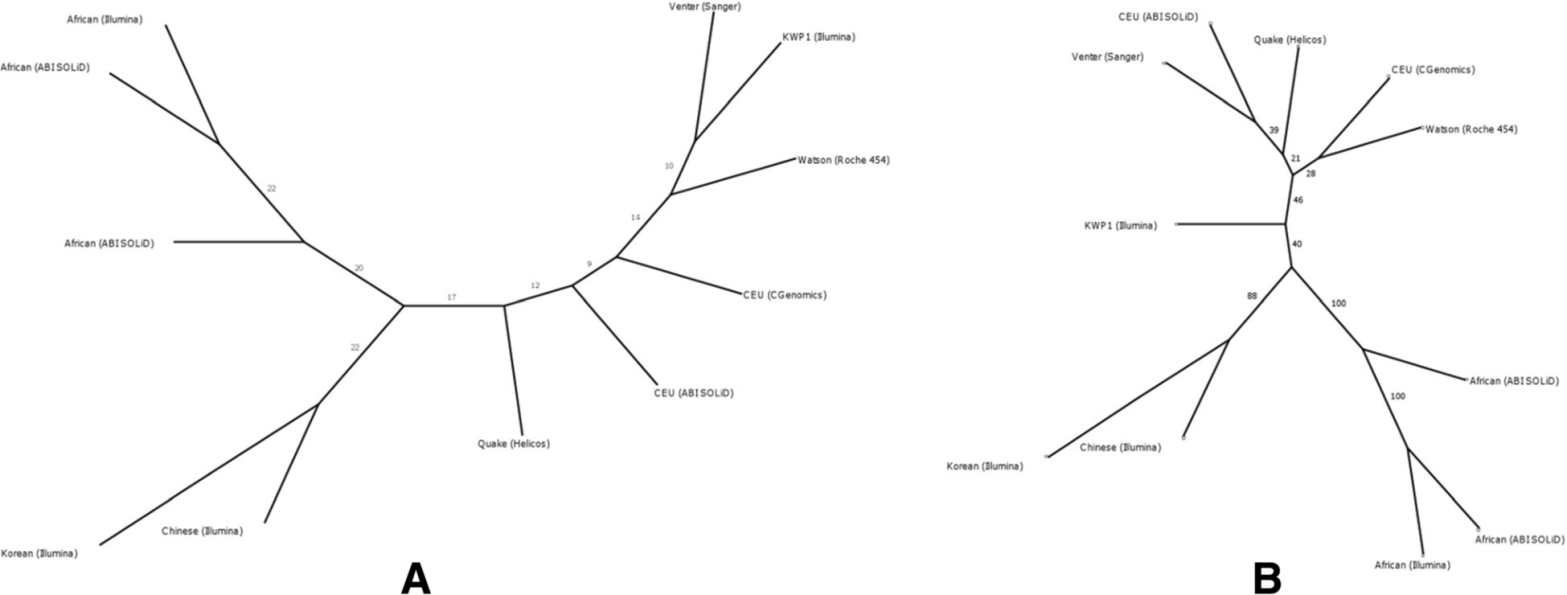
**Intergenome distances between the KWP1 genome and individuals from continental populations. (A)** Nearest-neighbor tree based on variant positions shared between the KWP1 samples and individuals from intercontinental populations. **(B)** Nearest-neighbor tree based on variant positions associated with OMIM disease genes and are shared between the KWP1 samples and individuals from intercontinental populations.

In order to assess the extent of variability that the KWP1 genome exhibits, we performed inter-genome comparisons with 10 representative genomes from four continents and derived nearest-neighbor tree based on genome-wide variant positions shared among genomes (Figure 2A). KWP1 genome is seen located in the vicinity of the five CEU genomes (Figure 2A). On rebuilding the tree using only those shared SNPs, which are previously associated with human diseases cataloged in OMIM database, we observe a change in the position of KWP1 genome, now lying between the Asian and the European genomes (Figure 2B) – this is in concordance with geographical location of the origin of sample i.e. at the nexus of Asian and European continents. This illustrates that disease profile of individual populations can be different, irrespective of their overall shared origin and that ethnicity acts as the dominant trend structuring disease-associated SNP locations.

### Annotation of the genome for structural variations

We identified 11,138 structural variations consisting of 7,645 deletions, 1,697 duplications, 585 insertions, 135 inversions and 1,076 translocations. Of the total 11,138 variations, as many as 10,283 (92.32%) are overlapping with known structural variations in DGV (Database of Genomic Variations, a curated catalog of human genomic structural variations) [46]. Furthermore, we see that 4279 (38.42%) of the reported structural variations lie in previously annotated human repeat-rich regions containing SINE (which include ALU), LINE and LTR repeat elements (Table 3).

**Table 3:** **Classification of identified structural variations**

**Type of structural variations**	**Number in Persian Genome**	**Reported in DGV**	**Reported to overlap with repeat rich regions in rmsk**
Deletions	7645	7190 (94.05%)	2969 (38.84%)
Duplications	1697	1575 (92.81%)	212 (12.49%)
Insertions	585	514 (87.86%)	362 (61.88%)
Inversions	135	104 (77.04%)	26 (19.26%)
Translocations	1076	900 (83.64%)	710 (65.98%)

A detected structural variation is defined to be ‘known’ if at least 50% of the detected variation (e.g. deletion) overlaps with a known variation.

### Concordance in SNP Calls between the deep sequencing experiment and genotyping experiment using Bead Chip arrays

**Figure 3:**
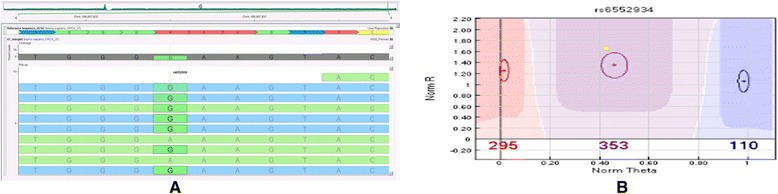
**Illustration of discordance in SNP calls between the deep sequencing experiment and genome-wide genotyping using bead chip arrays.** rs6552934 is considered as an example. **(A)** Sequencing data calls GG genotype. **(B)** Bead chip data calls AG genotype.

We observed an overall concordance of >99.8% for homozygous and heterozygous SNPs (homo- or heterozygosity is as per the genotype calls made in bead chip data) between sequencing and bead chip data; 190 of 194,862 heterozygous SNPs and 148 of 117,317 homozygous SNPs were seen wrongly called between two data sets. In 28 instances of the 190 mismatch heterozygous SNPs, they were called heterozygous in both data sets, but their alleles do not match., Of these 28 SNPs, 22 are annotated as multiallelic markers in dbSNP 138 and variant calls made using sequencing data are subsets of annotated alleles in dbSNP 138. Of the remaining 162 mismatch heterozygous SNPs, 62 lie in known copy number variation regions as annotated in DGV and 3 lie in known regions of genomic duplications as annotated in Genomic Super Dups database [47]. On examining the remaining 97 mismatch heterozygous SNPs, the read depth in these regions ranges from 2–35 with a mean depth of 14 (Additional file 13: Table S9). We looked at a SNP rs6552934, having a read depth of 9 with 7 reads supporting *G* allele and 2 reads supporting *A* allele, that is called as *GG* in sequencing data (Figure 3A) but as heterozygous *AG* in bead chip (Figure 3B); this instance of sequencing call clearly indicates that genotype calls using sequencing data can go wrong in regions of low coverage.

**Figure 4:**
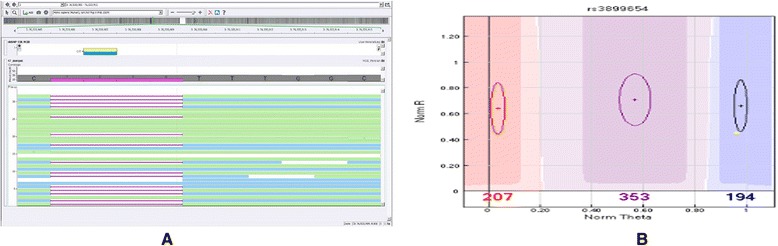
**Impact of novel SNPs and indels in the vicinity of SNPs typed on bead chip on genotype calling. (A)** Considered is an exemplary SNP of rs3899654, which has a novel heterozygous deletion of 2 bps upstream of the variant in KGP1 genome, that has CT call. **(B)** The typed marker in bead chip leads to inconsistent genotype call of CC.

**Figure 5:**
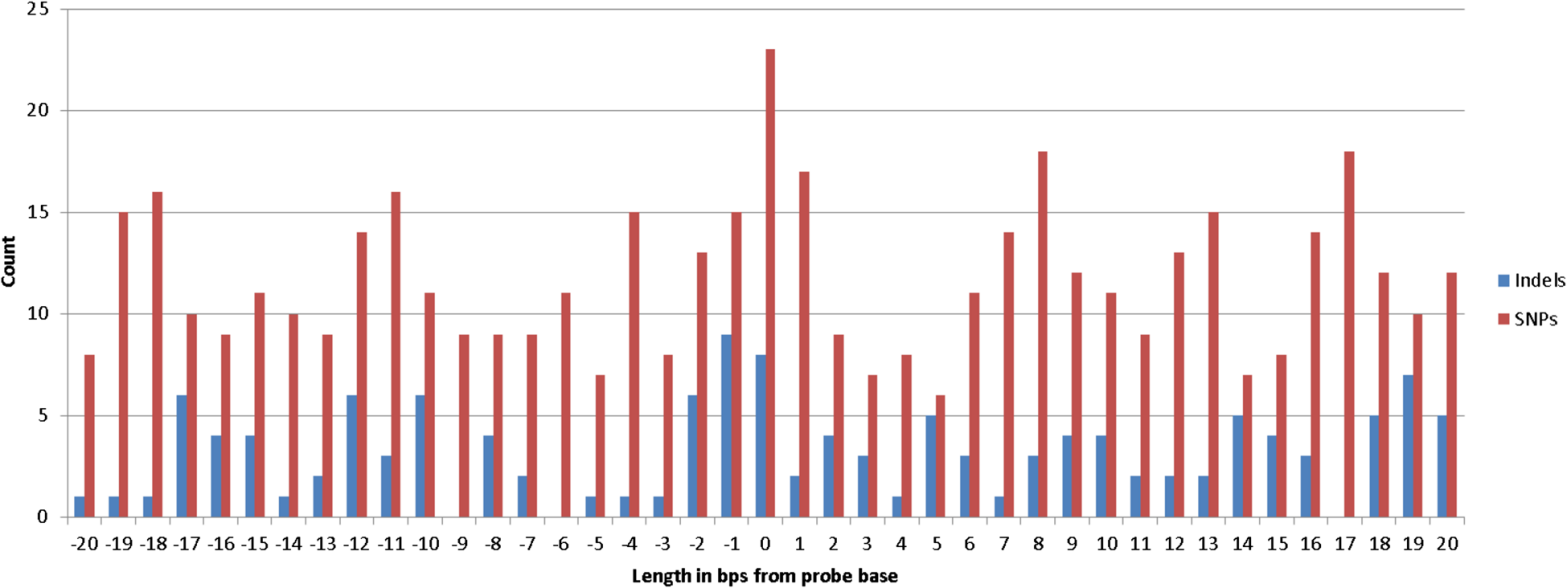
**Count of novel SNPs and indels in KWP1 genome around typed common markers in bead chips.**

Further, we examined the impact of novel SNPs and indels in the vicinity of SNPs typed on bead chip. We consider an exemplary SNP of rs3899654, which has a novel heterozygous deletion of 2 bps upstream of the variant in KGP1 genome, that is supposed to have *CT* call (Figure 4A); however, the typed marker in bead chip leads to inconsistent genotype call of *CC* (Figure 4B). Further, we illustrate that there exist a considerable number of novel SNPs and indels in KWP1 genome around the typed common markers (Figure 5) which can lead to inconsistent calls.

### Genome view of the variants

**Figure 6:**
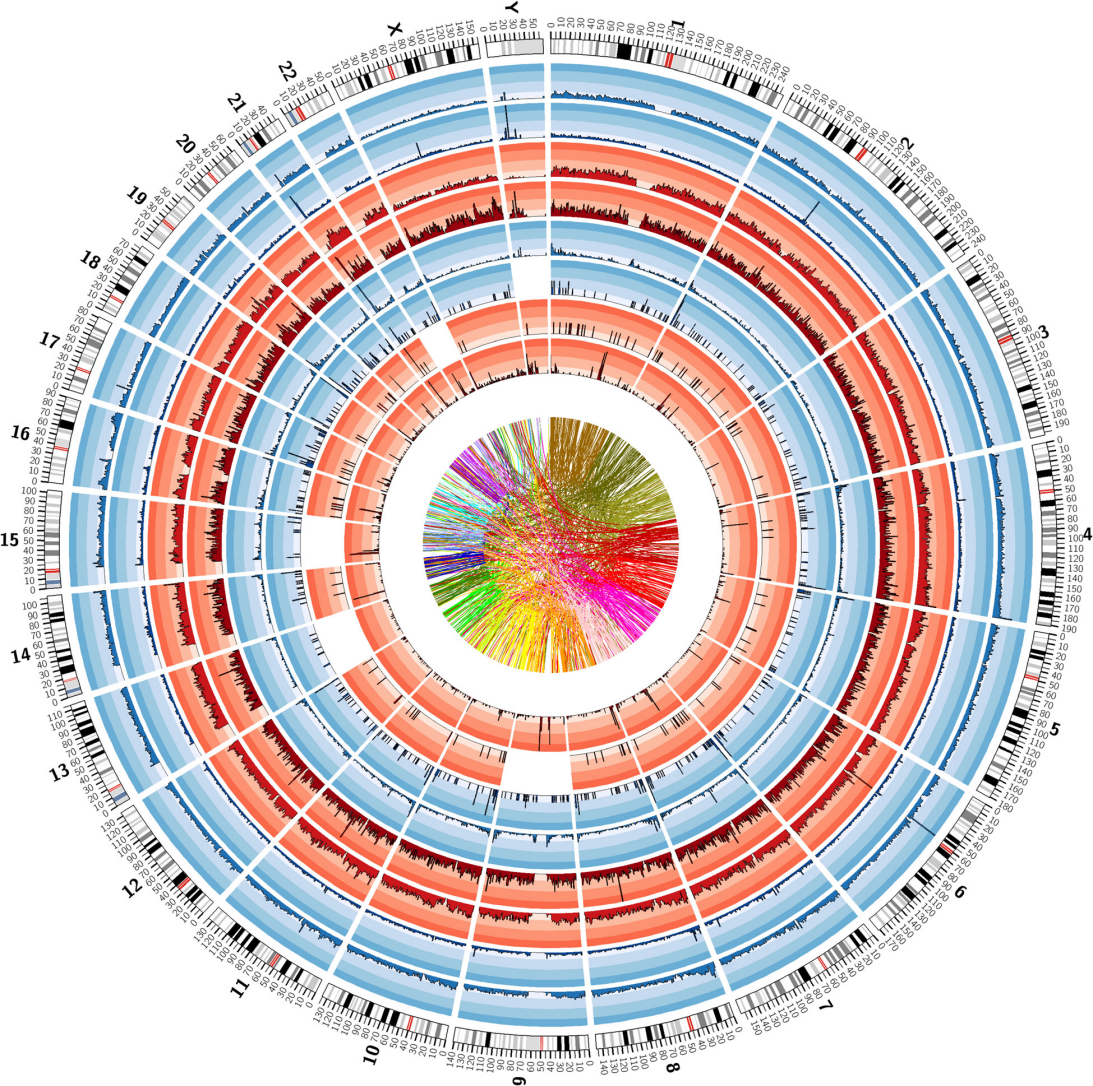
**Summary of analysis of genomes from Kuwait subgroup of Persian ancestry.** Tracks (from outer to inner): Karyotype of Human Genome; Density (in every window of 1 Mb) of ‘known’ SNPs (i.e. annotated in dbSNP 138); Density of ‘novel’ SNPs (i.e. not annotated in dbSNP138); Density of ‘known’ indels; Density of ‘novel’ indels; Density of Long Deletions; Density of Long Insertions; Density of Inversions; Density of Duplications; Links representing intra- and inter-chromosomal translocations. The image was generated using Circos [48].

Figure 6 provides a high-level view of the contents of the draft genome sequence in terms of density of known and novel variants (SNPs, short and long indels) observed in KWP1 genome, density of duplications, and the extent of chromosomal translocations. We have also created a genome browser (see the section on Availability of supporting data) for users to view an annotated display of the identified variants and structural variations, in the context of sequence and annotation tracks from other genome resources.

## Discussion

This study, as part of Kuwait Genome Project (that plans to derive reference genome sequences for the different subgroups of Kuwaiti population), analyzes genome of a Kuwaiti male participant with origin tracing back to Persian tribes of Iran. This separate genetic subgroup of Persian ancestry has also been reported in other states of Arabian Peninsula, such as Qatar [49]. We report around ~3.9 million genome variants, some of which are novel.

Presence of Persians in Kuwait dates back to seventeenth and eighteenth centuries, but due to absence of borders until the middle of twentieth century, there are no historical records available [50]. Myron push-pull theory [51] has often been used to explain Persian migration from southwest Iran to Kuwait. During the period of late nineteenth century to the early twentieth century, Southwest Iran was experiencing economic and political difficulties. These difficulties acted as push factors for Persian migration to places such as Dubai, Sharjah, Abu Dhabi, Al-Doha, Basrah and Manamah. In contrast, the pull factors typified by the political stability, economic prosperity, low taxes and low crime rate attracted Persians to State of Kuwait [52]. Presence of the genetic subgroup of Persian ancestry in other states of the Arabian Peninsula (as mentioned above) may suggest that migrations among Gulf States might also have happened; nautical trade along the Arabian Gulf might have facilitated such intra-Peninsula migrations. In modern-day Kuwait, up to 20 – 30% of the natives are of Persian descent [53].

Large-scale sequencing initiatives are being proposed [54] [http://rc.kfshrc.edu.sa/sgp/Index.asp] in the Peninsula with the aim to provide comprehensive data on genome variants instrumental in designing genome-wide bead arrays for conducting large-scale genetic studies in populations of the region. Bead arrays are designed with two assumptions: (1) the sequence in the sample is identical to the probe sequence, except at the position to be genotyped, and (2) there are only two possible alleles at the genotyped position for which the array is designed [55]. We find examples to show that falsifying any of the above two conditions can lead to wrong genotype calls from bead arrays. Previous reports on utilization of genome-wide bead arrays designed using reference human genome show that up to 34 % of published array-based GWAS studies for a variety of diseases utilize probes that overlap unanticipated SNPs, indels, or structural variants [56].

As sequencing experiments are gaining pace and large studies are being designed to decipher genetic relationships with common biological traits, unequal distribution of reads across genome regions can become a cause of concern. Previous reports have shown that differences in read depths across regions of genome and homozygous SNP calls flatten when the read depth reaches a threshold of 8, but heterozygous calls increase with increase in read depth [57,58]. In this paper, we show that a region of low read depth can cause false homozygous call using sequencing data, but correct heterozygous call was made using bead array. In a vicious circle of technology improvement, more large-scale sequencing studies on individual populations are needed to design more robust bead arrays, however these large sequencing projects can utilize the current bead arrays to improve genotype calling in low coverage regions.

We demonstrate that the genome of the sequenced individual, who suffers from Type 2 diabetes, harbors as many as 72 (out of the 159) variants previously associated with Type 2 diabetes. This observation has implications on global initiatives that strive to develop genome panels to help in genetic susceptibility testing for Type 2 diabetes [59,60]. Several commercial companies offer genome panels incorporating up to 38 variants designed for different ethnicities [61]. Clinical utility evidence provided by such panels that include differing number of variants up to 38 has been found to be inadequate. In our current study, we find that up to 72 markers relating to Type 2 diabetes are seen in the sequenced genome.

We further demonstrate that the presented genome, of the individual who has a medical history of β-Thalassemia (Minor), harbors three of the four β-Thalassemia variants annotated in GWAS Catalog. Thalassemia and Sickle cell anemia are the most prevalent genetic blood diseases in Kuwait [62,63]. Furthermore, it is known that β-thalassemia is the most common hereditary disease in Iran [64]; the origin of the individual sequenced in this study traces back to the Fars province of Iran.

It is further assessed that the family of the individual sequenced in this study has a history of migraine. In concordance with this medical history, we find that the genome of the individual harbors 76 (out of 169) variants that are associated with migraine in GWAS Catalog.

The identified variants include deleterious and loss-of-function coding variants, as well as critical non-coding variants that are associated with phenotype traits – some of the notable phenotype traits include: decrease in birth weight, protection towards Type 1 diabetes, decrease in long term memory, greater triglyceride lowering ability in response to fenofibrate treatment, and higher warfarin doses to elicit anticoagulation response.

## Conclusions

This is the first study to report a reference genome resource for the population of Persian ancestry. We report novel genome variants that include SNPs, indels and structural variations that enlarge the current repertoire of human genome variation. Nearest-neighbor tree built using shared disease-causing variants between the Persian genome and other continental genome positions the Persian genome between the Asian and European genomes; this is in concordance with the geographical location of the origin of the sample at the nexus of Asian and European continent. Apart from the findings from population-context, the study illustrates that the participant’s medical history of Type 2 diabetes and β-Thalassemia as well as the medical history of migraine in the family of the participant, are accounted by the presence of a large number of genome variants that are known to be associated with these traits. The presented genome data provides a starting point for designing large-scale genetic studies in Persian population.

## Methods

### Ethics statement

The study was approved by the Scientific Advisory Board and the Ethics Advisory Committee at Dasman Diabetes Institute, Kuwait. Written informed consent for the study was obtained from the participant before blood samples were collected.

### Sample collection and library preparation

Blood sample was collected in EDTA 4 ml tube. Gentra Puregene® kit (Qiagen, Valencia, CA, USA) was used to extract DNA as per manufacturer’s protocols. DNA was quantified using both the Quant-iT™ PicoGreen® dsDNA Assay Kit (Life Technologies, NY, USA) and the Epoch Microplate Spectrophotometer.

Prior to library preparation, frozen DNA stock was diluted to a working solution of 50 ng/μl as recommended by Illumina (CA, USA) and was quantified by agarose gel analysis. DNA sample was sheared to generate uniform size segments averaging around 400 bps by using Covaris E220 instrument (Covaris, Woburn, MA, USA) with the parameters of Duty Cycle 10%; Intensity 4; Cycles per Burst 200; and Time 55 seconds. Sheared DNA was used to prepare sequencing libraries using TruSeq DNA sample preparation and cBot Paired End (PE) cluster generation kits as per manufacturer’s protocols (Illumina, CA, USA). Libraries were loaded on a HiSeq 2000 flow cell for paired-end sequencing using the TruSeq SBS 200 cycles chemistry

### Image analysis and alignment of reads from whole genome sequencing

CASAVA v1.8.2 (Illumina Inc, USA) was used for demultiplexing and Bcl to fastq conversion. The generated paired-end reads were aligned to human reference genome hg19 using BWA v0.6.2 [65]; default parameters were used with the exception of “–q 30” for aln command to allow trimming at the 3′ ends of the reads. The resultant SAM files were converted to BAM using Sequence Alignment/Map (SAM) tools v0.1.18 [66], where bit representing “each segment properly aligned according to the aligner” is set in SAM bitwise FLAG. Alignments were visualized using GenomeBrowse™ v1.1 by Golden Helix, Inc.

### SNP and indel discovery

We used a modified HugeSeq [67] pipeline, which enables the pipeline to run on multi-core Linux Desktops, to standardize the variant discovery process. Quality control checks on BAM files were performed before variant calling using tools in Genome Analysis Toolkit (GATK) v2.4-7-g5e89f01 [68] and Picard v1.86 toolkit as recommended in HugeSeq pipeline. We observe 4,118,585 variants (SNPs + indels) using GATK UnifiedGenotyper [20] and 4,227,839 variants using SAMtools. The consensus set between the two methods, as reported in this work, includes 3,977,923 variants. The consensus set, which was determined using vcftools isec method [69], was used in all subsequent analyses.

### Mitochondrial and Y-chromosome haplogroup analysis

The paired-end reads aligned to hg19 mitochondrial sequence were realigned to rCRS (Revised Cambridge Reference Sequence) [70] using all the quality control steps that we adopted for calling variants. The variants were used to call haplogroups using HaploGrep [71]. The data conversion from VCF file to HaploGrep input file (.hsd file) was performed manually. The Y-chromosome variants were used to call haplogroups using AMY-tree software [72].

### Annotation of variants

SNP & Variation Suite v8.1 (SVS) [Bozeman, MT: Golden Helix, Inc] was used to annotate variants as “known” or “novel” by considering dbSNP 138 database as reference.; a variant is annotated as “novel”, if the variant is not present in dbSNP or the alternate allele seen in the genome is not a subset of those annotated in dbSNP database. We further classified the variants according to their genomic annotation into 10 categories using the SVS tool. Exonic variants were further classified into 13 categories based on their location within the genes and their effects on protein sequences. The nonsynonymous variants were examined for damaging effects on the encoded proteins by using SIFT [73] and PolyPhen2 [74] annotations. Variants that are annotated either by SIFT as ‘Damaging’ OR by PolyPhen2 as ‘Probably Damaging’ are classified as “deleterious” [75]. The NHGRI GWAS Catalog [76] was used to annotate SNPs for association with human diseases and other phenotype traits using Annovar [77].

### Detecting structural variations

We used four different algorithms as part of HugeSeq pipeline, to detect structural variations from paired-end reads. BreakDancer version 1.1 [78] uses anonymous long or short span size between the paired-end reads to identify long indels. Furthermore, it identifies both intra- and inter-chromosomal translocations. Pindel version0.2.4 [79] was used for split‐read analysis. CNVnator version 0.2.7 [80] was used to perform read‐depth analysis. BreakSeq Lite version 1.0 [81] was used for junction mapping. Calls from the different tools were merged, using BEDTools [82], if the reciprocator overlap between two variants is ≥ 0.5. Deletions were annotated using Annovar [77]. A detected structural variation is defined to be ‘known’ if at least 50% of the detected variation (e.g. deletion) overlaps with annotated variations (e.g. deletions) in the Database of Genomic Variants [46,83]; otherwise, the deletion is considered to be “novel”. Repeat content of the identified structural variations are estimated using rmsk (RepeatMasker) tables from UCSC [84].

### Calculation of intergenome distances between the KWP1 genome and representative genomes from continental populations

We consider a total of 10 genomes covering diverse ethnicities (African, Asian, European, and American), downloaded from the sites of 10Gen [85], for comparing the intergenome similarities with the genome sequenced in our study.

We adopted the methods developed by Moore et al. [85] to calculate intergenome distances based on information relating to shared variant locations between genomes. We constructed the nearest-neighbor tree that is based on such calculated intergenome distances using PHYLIP [86].

Similar method was used to construct phylogenetic tree using 100 Qatari exomes [13] without bootstrap analysis.

### Depiction of consensus nearest-neighbor tree based on shared variants among genomes with known disease-causing/predisposing alleles as cataloged in OMIM

The tree depicting OMIM phylogenetic comparison is constructed with the method of Moore et al. (described in the previous section) used to construct the nearest-neighbor tree based on genome-wide shared variants, but restricting the shared variant locations between genomes to include only those locations where at least one of the genomes contain an OMIM [87] allele. The tree was bootstrapped 50 times, and labels on the nodes depict the resulting bootstraps.

### Building the genome browser to visualize the sequenced genome

Genome browser provides an effective means to share genome data to biomedical community; we have set up JBrowse (version 1.8.1), a graphical interface to enable access to the reported whole genome sequences. JBrowse is an open-source project for genome browser [88]. We have facilitated visualization of external data (such as genome variants reported in Database of Genomic Variant and dbSNP 138) along with the KWP1 genome.

### Availability of supporting data

The reported whole genome sequence and all the identified variants (known and novel) are available on the ftp site (http://dgr.dasmaninstitute.org/DGR/downloads.html). The data can be visualized using genome browser with other annotations tracks from UCSC at http://dgr.dasmaninstitute.org/DGR/gb.html. Proper functionality of the web server requires Firefox version 6 (or later versions) or Internet Explorer version 10 (or later versions).

In addition, the data has been deposited with the NCBI data repositories. The whole genome sequencing reads are deposited with NCBI SRA (Sequence Read Archive) (accession number: SRX535330), the SNPs and indels with NCBI dbSNP (Database of short genetic variations), and the CNV/SV structural variation calls with NCBI dbVar (Database of genomic structural variation) (accession number: nstd99).

## Additional files

Additional file 1: Figure S1. PCA depicting position of sequenced sample in three Kuwaiti population subgroups. Additional file 2: Figure S2. Nearest-neighbor tree based on variant positions shared between the KWP1 samples and 100 exomes from Qatar. QA: Qatari Exomes of Sub-Saharan African Ancestry; QB: Qatari Exomes of Bedouin Ancestry; QP: Qatari Exomes of Persian Ancestry. Additional file 3: Figure S3. Chromosomal distribution of mean Ti/Tv ratio for known and novel markers. Additional file 4: Table S1. Classification of the identified SNPs based on genome annotation. SNP & Variation Suite v8.1 (SVS) [Bozeman, MT: Golden Helix, Inc] was used to annotate variants. Additional file 5: Table S2. List of the 61 identified exonic LOF (Loss of Function) SNPs that are homozygous alternate. These SNPs lead to premature stops and splice-site disruptions resulting in complete loss of function. Additional file 6: Table S3. List of the 69 identified exonic LOF (Loss of Function) indels that are homozygous alternate. These indels lead to premature stops, splice-site disruptions and frameshifts resulting in complete loss of function. Additional file 7: Figure S4. Allele frequency analyses of the identified homozygous exonic Loss-of-Function SNPs. Allele frequencies were obtained using data from the 1000 Genomes Project. Additional file 8: Table S4. List of 17 LOF SNPs annotated as expression quantitative trait loci (eQTLs) in various populations. This list of 17 SNPs is a subset of the 61 LOF homozygous alternate SNPs presented in Additional file 5: Table S2. Additional file 9: Table S5. List of the 2,123 ‘known’ deleterious SNPs identified in the Persian genome sequenced in this study. Variants that are annotated either by SIFT as ‘Damaging’ OR by PolyPhen2 as ‘Probably Damaging’ are classified as “deleterious”. Additional file 10: Table S6. List of the 91 ‘novel’ deleterious SNPs identified in the Persian genome sequenced in this study. Variants that are annotated either by SIFT as ‘Damaging’ OR by PolyPhen2 as ‘Probably Damaging’ are classified as “deleterious”. Additional file 11: Table S7. Genotype-phenotype associations (as annotated in NHGRI GWAS Catalog) in the case of 6,328 ‘known’ non-coding SNPs identified in the Persian genome sequenced in this study. These SNPs are associated with 811 traits. Additional file 12: Table S8. Genome wide association studies annotating the 72 Type 2 Diabetes (T2D) SNPs identified in the Persian genome sequenced in this study. This list of 72 T2D SNPs is a subset of 159 T2D SNPs annotated in NHGRI GWAS Catalog. Additional file 13: Table S9. Read depth in genome regions containing heterozygous SNPs with genotype calls that are discordant between sequencing and bead chip data. On examining 97 such heterozygous SNPs, the read depth in these regions ranges from 2–35 with a mean depth of 14.
