# Supplementary figures and images for: Sequence and analysis of a whole genome from Kuwaiti population subgroup of Persian ancestry
Source: BMC Genomics. 2015 Feb 18;16(1):92. doi: 10.1186/s12864-015-1233-x (PMC4336699; doi:10.1186/s12864-015-1233-x)

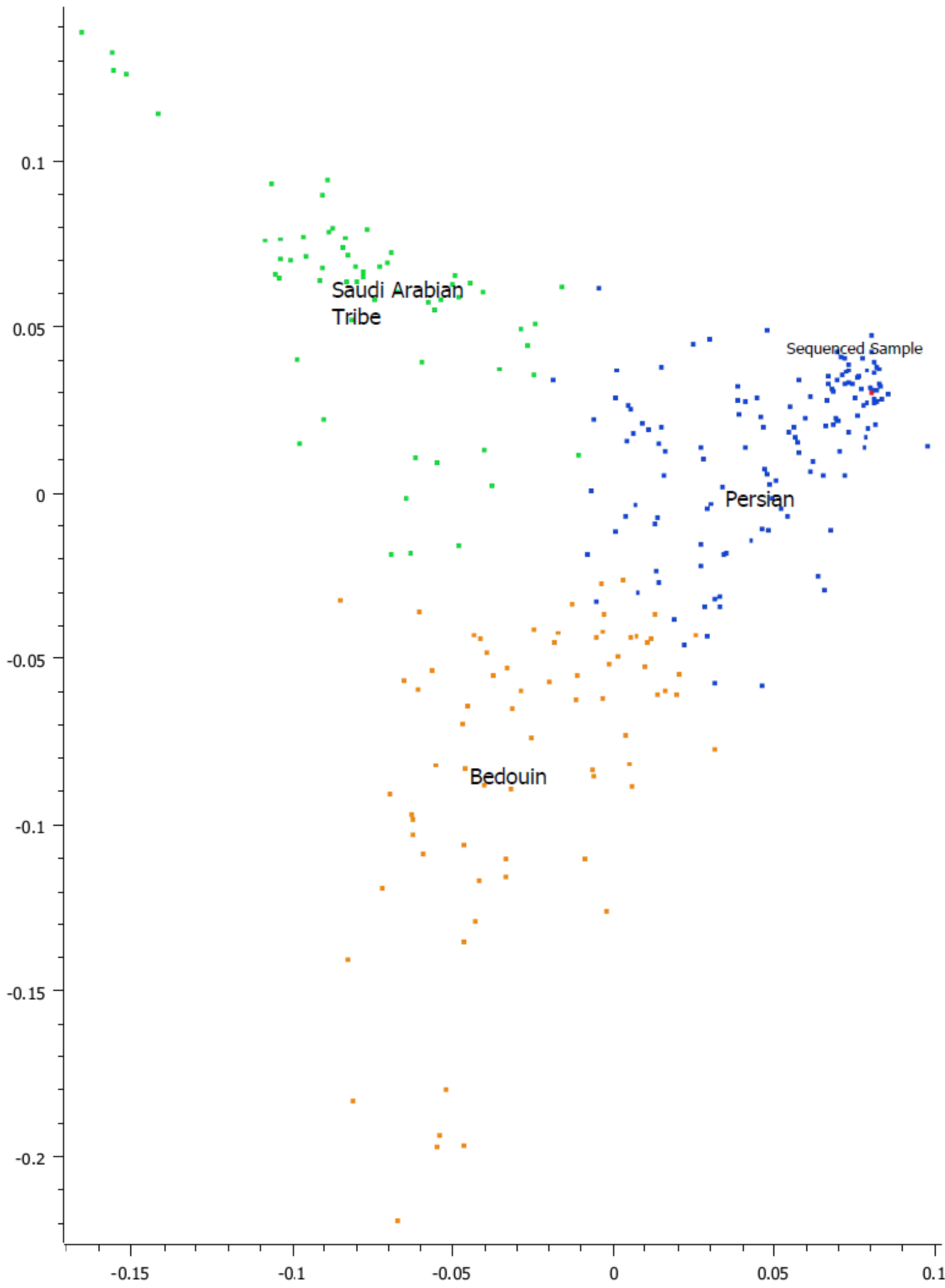

Supplement: Additional file 1: Figure S1. — PCA depicting position of sequenced sample in three Kuwaiti population subgroups. [file 12864_2015_1233_MOESM1_ESM.pdf]

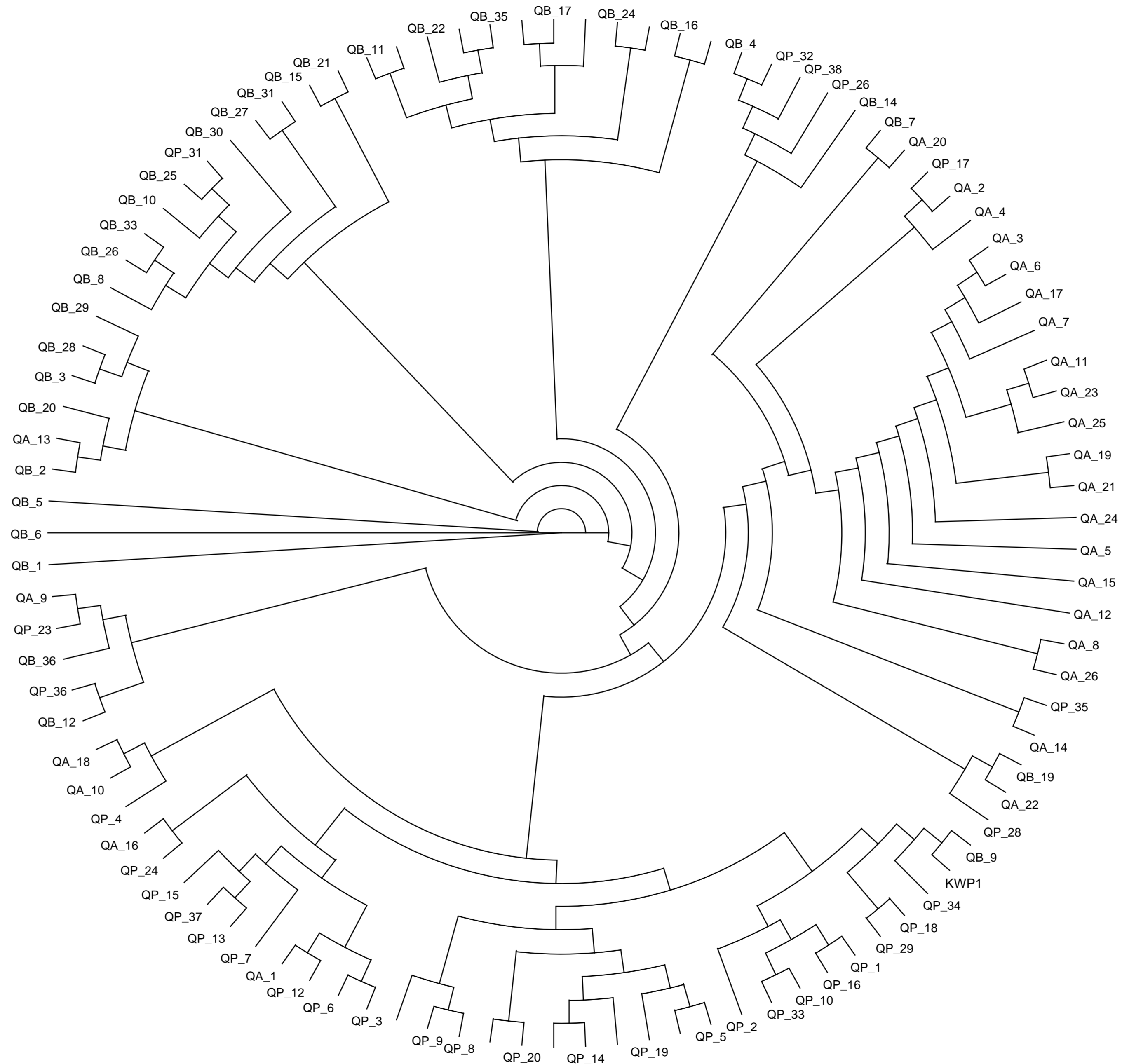

Supplement: Additional file 2: Figure S2. — Nearest-neighbor tree based on variant positions shared between the KWP1 samples and 100 exomes from Qatar. QA: Qatari Exomes of Sub-Saharan African Ancestry; QB: Qatari Exomes of Bedouin Ancestry; QP: Qatari Exomes of Persian Ancestry. [file 12864_2015_1233_MOESM2_ESM.pdf]

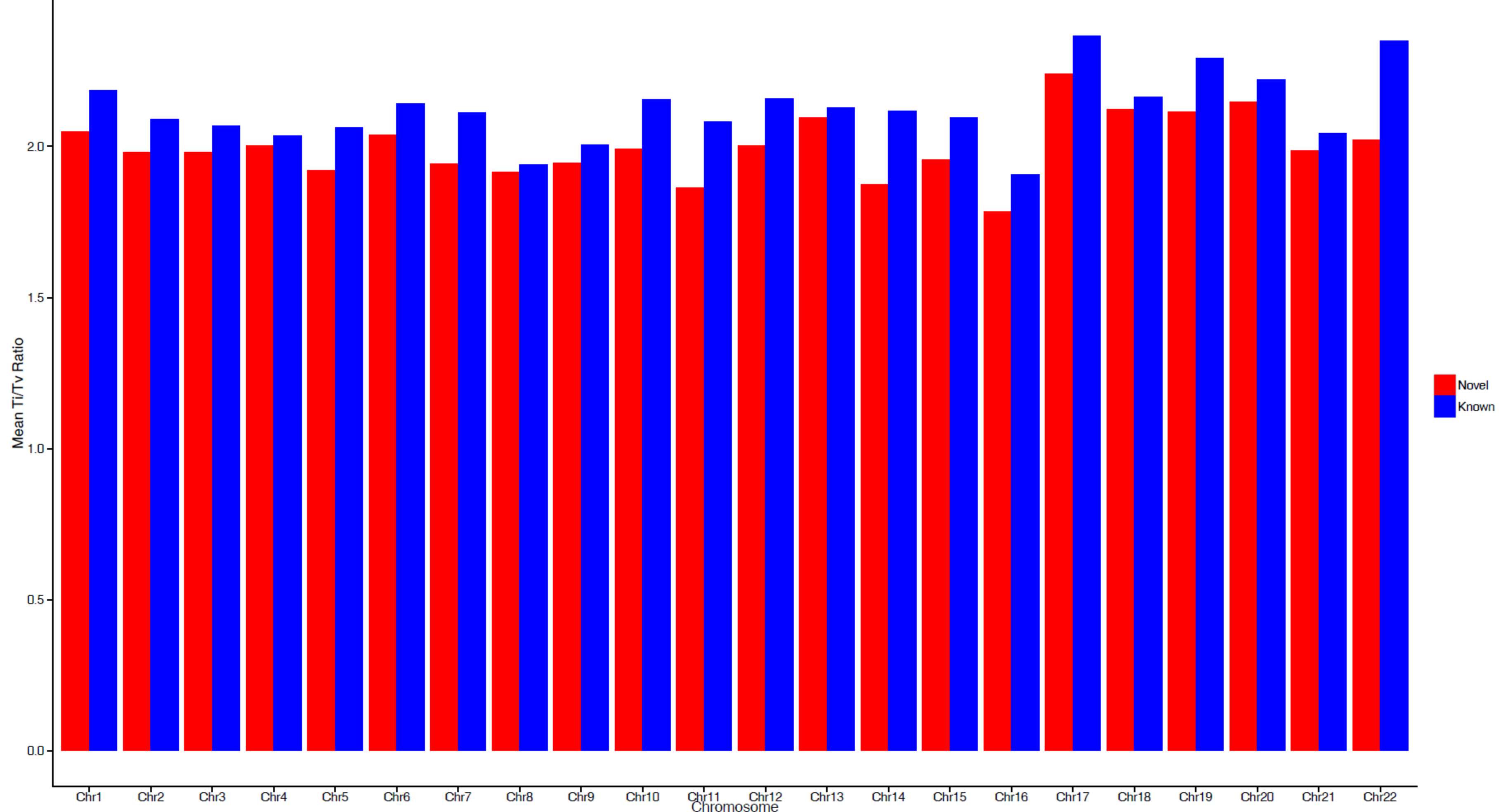

Supplement: Additional file 3: Figure S3. — Chromosomal distribution of mean Ti/Tv ratio for known and novel markers. [file 12864_2015_1233_MOESM3_ESM.pdf]

▪ All Indiv Freq

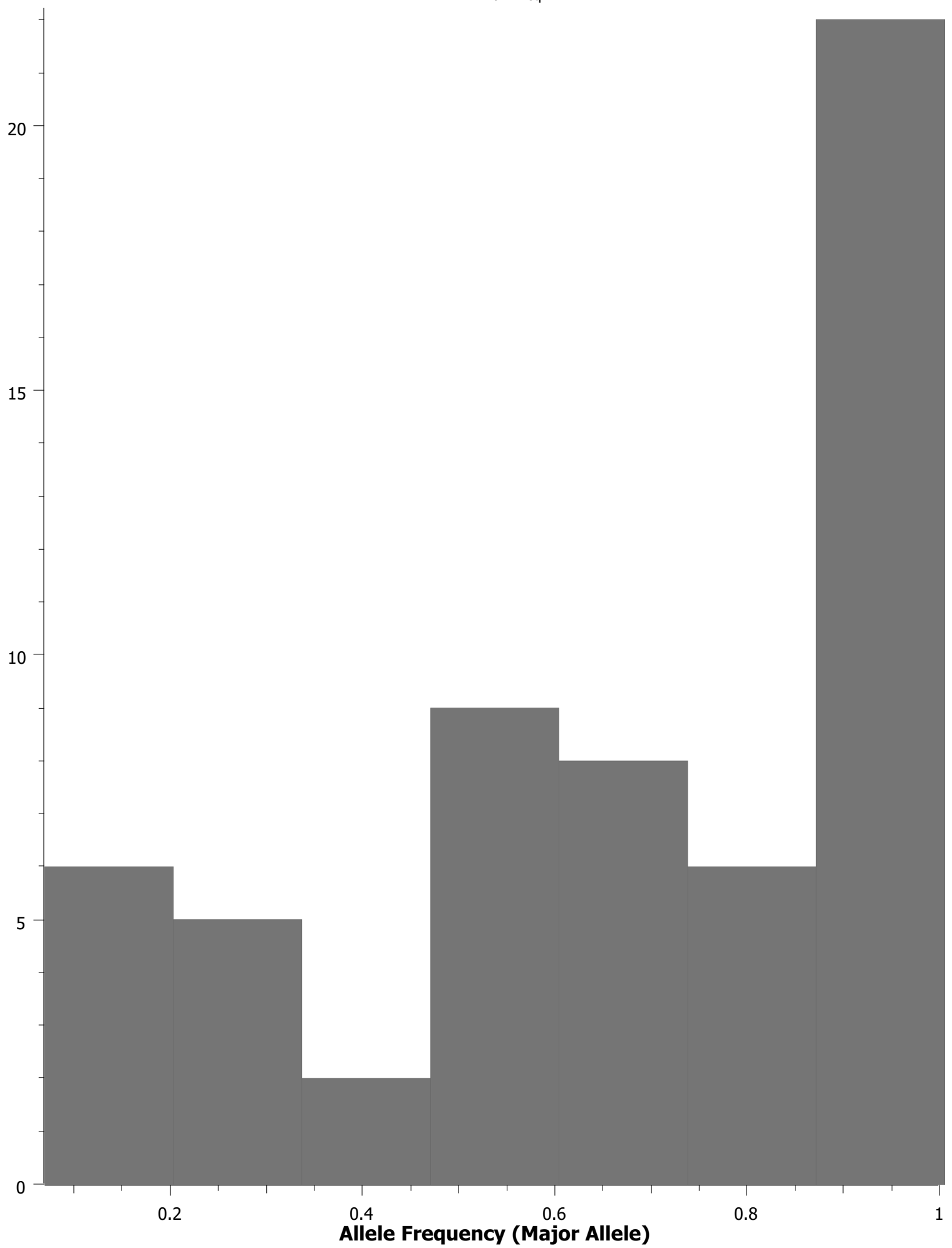

Supplement: Additional file 7: Figure S4. — Allele frequency analyses of the identified homozygous exonic Loss-of-Function SNPs. Allele frequencies were obtained using data from the 1000 Genomes Project. [file 12864_2015_1233_MOESM7_ESM.pdf]
